# Different axis approaches for ultrasound-guided centrally inserted central catheterization in children: a systematic review and meta-analysis of randomized controlled trials

**DOI:** 10.3389/fsurg.2025.1481975

**Published:** 2025-02-24

**Authors:** In Kyung Lee, Kyeong Hun Lee, Hye-ji Han, Jieun Choi, Na Jin Kim, Kyunghoon Kim

**Affiliations:** ^1^ Department of Pediatrics, Seoul St. Mary’s Hospital, Seoul, Republic of Korea; ^2^ Department of Pediatrics, College of Medicine, The Catholic University of Korea, Seoul, Republic of Korea; ^3^ Department of Pediatrics, Seoul National University Bundang Hospital, Seongnam, Republic of Korea; ^4^ Medical Library, The Catholic University of Korea, Seoul, Republic of Korea; ^5^ Department of Pediatrics, Seoul National University College of Medicine, Seoul, Republic of Korea

**Keywords:** central venous catheters, vascular access, ultrasonography, pediatrics, meta-analysis

## Abstract

**Background:**

Centrally inserted central catheterization (CICC) is a critical procedure in pediatric care. However, CICC in children poses greater challenges compared to adults due to anatomical and physiological differences, leading to higher complication rates. Ultrasound-guided approaches have been developed to enhance the safety and effectiveness of CICC, but the comparative efficacy of different axis approaches remains unclear.

**Methods:**

A systematic review and meta-analysis of randomized controlled trials comparing different axis approaches for ultrasound-guided CICC in children was conducted. Searches were carried out in databases up to June 10, 2024. Six studies were included in the systematic review and three studies were included in the meta-analysis. Primary outcomes included first-attempt success rate, overall success rate, and cannulation time. Secondary outcomes were complications such as hematoma and posterior wall puncture.

**Results:**

Data from 547 children were analyzed. The long-axis in-plane approach significantly reduced cannulation time (MD −27.48 s, 95% CI, −33.99 to −20.97) and overall complications OR 0.21, 95% CI, 0.1–0.48) compared to short-axis out-of-plane approach. No significant differences were found in first-attempt or overall success rates between the long-axis and short-axis approaches.

**Conclusion:**

The long-axis approach for ultrasound-guided CICC in children offers significant advantages in reducing cannulation time and complications. While dynamic needle tip positioning method may serve as an alternative to in-plane methods, further studies are needed to validate its clinical efficacy. Further research is needed to refine these techniques and explore their application in diverse clinical settings.

## 1 Introduction

Central catheter placement is crucial for critically ill patients requiring hemodynamic monitoring, and vasoactive drug administration (1, 2). However, centrally inserted central catheterization (CICC) in children presents more challenges and higher complication rates compared to adults (3, 4). The success and risk of complications also depend on the patient's condition, anatomy, and the operator's skill (4–6). Therefore, it is essential to identify a safe and effective method for CICC in children.

In pediatric patients, CICC is generally performed through the internal jugular, subclavian, or femoral vein (7). Traditionally, this procedure used anatomical landmarks and techniques such as the Seldinger technique (8, 9). With the advent of ultrasound (US) in intensive care settings, US-guided approaches have gained prevalence (10). Compared to anatomical approaches, US-guided CICCs have shown higher success rates, fewer puncture attempts, and reduced complication rates (11–14).

The US probe for central venous access can be oriented to provide either a “short-axis” (cross-sectional view) or a “long-axis” (longitudinal view) image of the vessel (15, 16). Needle insertions are classified as in-plane or out-of-plane based on their visibility in the US image (15). A combined technique begins with a short-axis view and then rotates the probe to a long-axis view (17). The modified dynamic needle tip positioning (DNTP) is a modified short-axis out-of-plane technique designed to improve needle tip tracking (18). Unlike the traditional static short-axis out-of-plane approach, where the transducer remains fixed and the needle is advanced blindly, DNTP involves dynamic transducer movement to intermittently relocate the needle tip.

The comparative effectiveness and safety of the short-axis out-of-plane vs. long-axis in-plane approaches have not been conclusively established in adult patients (19–21). This study evaluates the effectiveness and complications associated with the short-axis out-of-plane, long-axis in-plane, DNTP methods for US-guided CICC in children through a systematic review and meta-analysis.

## 2 Methods

### 2.1 Study design

A systematic review of RCTs comparing different axis approaches for ultrasound-guided CICC in children was conducted, accompanied by a meta-analysis to evaluate the effectiveness and safety of these CICC approaches. The study adhered to the Preferred Reporting Items for Systematic Reviews and Meta-Analyses (PRISMA) guidelines (22).

### 2.2 Database and search strategy

A comprehensive and peer-reviewed search strategy was developed by a medical librarian (NJK). Searches were carried out in PubMed, Embase, and The Cochrane Library from inception to June 10, 2024, employing terms related to US, CICC, and pediatric age. The detailed search strategy is outlined in Supplementary 1.

### 2.3 Data collection and analysis

Two independent reviewers (IKL and KHL) screened titles and abstracts to identify potentially eligible trials. They then evaluated the full texts of the selected studies for eligibility. Any discrepancies between the reviewers were resolved through discussion, and if necessary, a third reviewer was consulted to reach a consensus.

### 2.4 Inclusion criteria

Included were trials that: (1) involved studies with children; (2) were RCT; and (3) compared different axis approaches for US-guided CICC.

### 2.5 Exclusion criteria

Excluded were trials that: (1) were observational studies, case reports, letters, editorials, or were not peer -reviewed; (2) included duplicate samples; (3) involved only adult participants; (4) did not utilize US for CICC; or (5) involved studies with peripherally inserted central catheters.

### 2.6 Outcomes

The primary outcome focused on catheterization success rates and cannulation time. Secondary outcomes included complications such as overall complication, hematoma, and posterior wall puncture.

### 2.7 Quality assessment

The risk of bias in included trials was assessed by two reviewers (IKL and KHL) using a modified version of the Cochrane risk of bias tool (23). Each trial was examined for bias across various domains, with each domain assessed as having low, unclear, or high risk. The classification of the overall risk of bias for each trial was as follows: classified as low if the risk of bias was low or possibly low in all domains, classified as unclear if there was an unclear risk of bias in at least one domain with no domain having a high risk of bias, and classified as high if there was a high or possibly high risk of bias in any domain. Any discrepancies were resolved through discussion and consensus.

### 2.8 Statistical analysis

The meta-analysis employed R version 4.2.2 (R Foundation for Statistical Computing, Vienna, Austria) to analyze different axis approaches of CICC. For continuous outcome data, the mean difference served as the primary measure, with estimates aggregated using the inverse variance method. The Mantel-Haenszel method pooled estimates for binary outcome data, using odds ratio and risk ratio as primary metrics. The choice between a common or a random effects model was based on heterogeneity levels, indicated by *I*^2^ exceeding 50%, favoring a random effects model at that point.

## 3 Results

### 3.1 Study selection and characteristics

**Figure 1:**
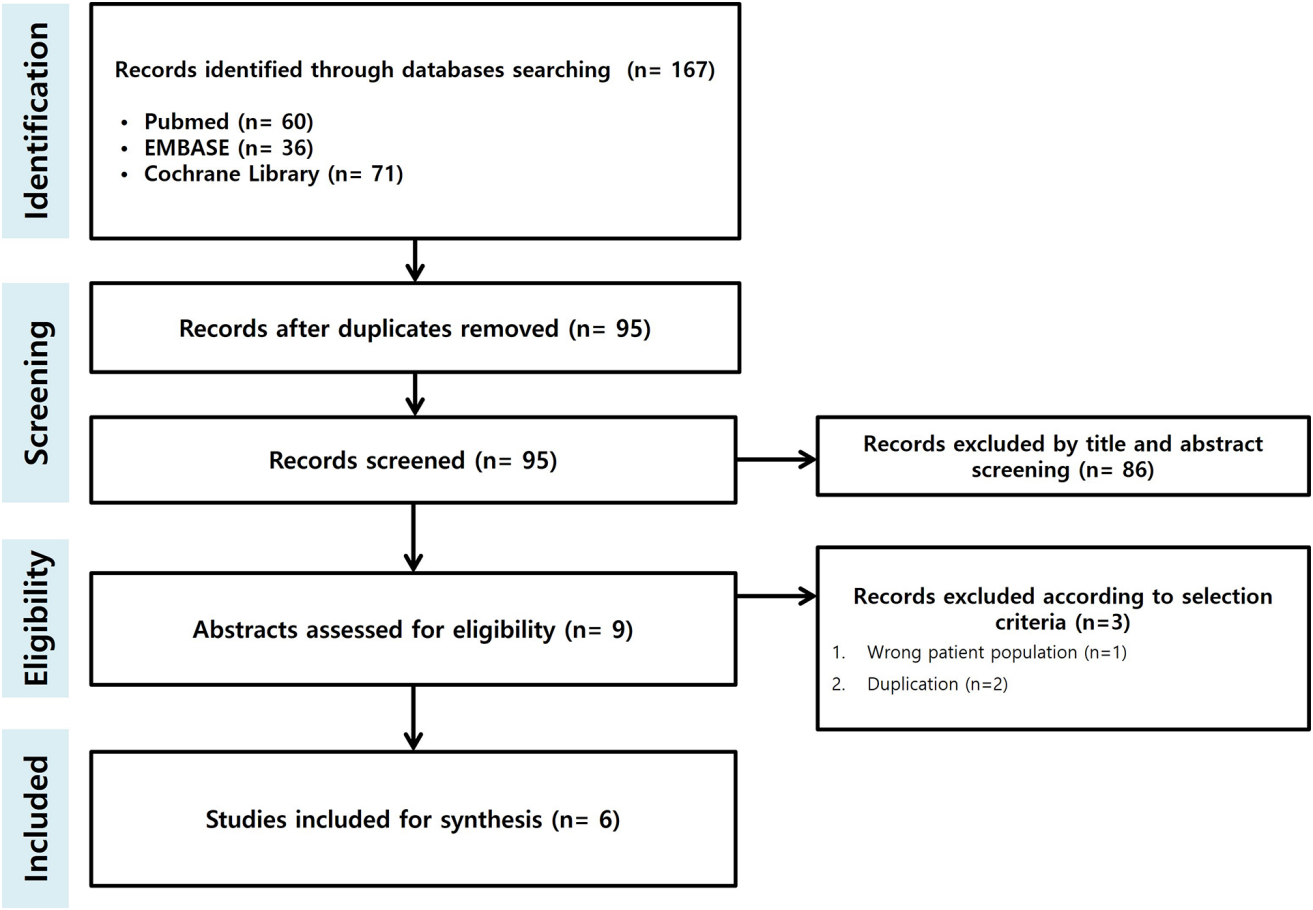
Flowchart illustrating the study selection process.

The search identified a total of 167 records. After screening and assessing for eligibility, six studies were included in the systematic review, and three studies were included in the meta-analysis (24–29) (Figure 1).

Among these, two studies focused on neonates, and four studies included participants scheduled for surgery. Three studies compared the long-axis and short-axis approaches, while two studies compared the DNTP approach to conventional approaches, such as the long-axis or combined short- and long-axis approaches (Table 1). However, studies including DNTP approaches were not included in the meta-analysis due to heterogenous comparison groups.

**Table 1:** Characteristics of included studies.

Author, year	Study period	Inclusion criteria	Exclusion criteria	Intervention	Control	Number of patients (I/C)	Catheterization site	Outcomes
Liu, 2020 (24)	No information available	Newborns with a gestational age of less than 37 weeks scheduled to undergo surgery requiring CICC	Malformation of the neck, skin infection or damage in the puncture area, deep vein thrombosis on ultrasound, history of internal jugular vein catheterization, or parents who refused to participate in this trial	Combined short- and long-axis	Short-axis DNTP	90 (45/45)	Internal jugular vein	First-attempt success rate, total success rate, procedure time, and number of failed attempts
Takeshita, 2020 (25)	April 2019∼Dec 2019	Children less than 5 years old who required CICC for perioperative management of cardiovascular surgery	Patients who underwent emergency surgery or in whom the CICC had already been inserted	Long-axis	Short-axis	97 (49/48)	Internal jugular vein	Posterior wall puncture, first attempt success, overall success, procedure duration, number of attempts
Keskin, 2021 (26)	No information available	Children aged 3 months to 15 years, who had been admitted to PICU, and had an indication for CICC	Patients younger than three months and older than 15 years, those weighing <5,000 g, those with any anatomic malformation in the neck, infection at the intervention site, or thrombosis detected by US, those with a history of internal jugular vein catheterization, and those whose parents did not provide consent for participation	Syringe-free, long-axis	Short-axis	60 (30/30)	Right jugular vein	Performing time, first-pass success, number of needle passes, number of skin punctures, complications, hematoma, carotid puncture, posterior wall puncture
Tan, 2022 (27)	Nov 2018∼Oct 2019	Neonates scheduled to undergo cardiothoracic, general, or neurosurgery requiring CICC	Skin erosions or hematomas at or near the insertion site, visible recent catheterization scars, or any thrombotic formations within the vein	Long-axis	Modified DNTP short-axis	90 (45/45)	Internal jugular vein	Cannulation time, first-attempt success rate, total success rate, hematoma, common carotid artery puncture, pneumothorax, CRBSI
Takeshita, 2022 (28)	Feb 2020∼Jan 2021	Aged <5 years who underwent cardiovascular surgeries and required CICC	Emergency surgery	Combined short- and long-axis	Long-axis	110 (55/55)	Internal jugular vein	Posterior wall puncture, first attempt success, overall success, number of attempts, scanning duration, puncture duration, total procedure duration
Kumar, 2023 (29)	June 2020∼June 2022	ASA Physical status I/II pediatric patients aged 0–1 year old scheduled for CICC	ASA III or more, patients with anatomical abnormality at the clavicular region, local infections at the supraclavicular region, and obesity	Long-axis	Short-axis	100 (50/50)	Brachiocephalic vein	First-attempt success rate, overall success rate, the number of attempts, and cannulation time/performance time

CICC, centrally inserted central catheterization; DNTP, dynamic needle tip positioning; CRBSI, catheter-related bloodstream infection; PICU, pediatric intensive care unit; US, ultrasound; ASA, American Society of Anesthesiologist.

### 3.2 Risk of bias

**Figure 2:**
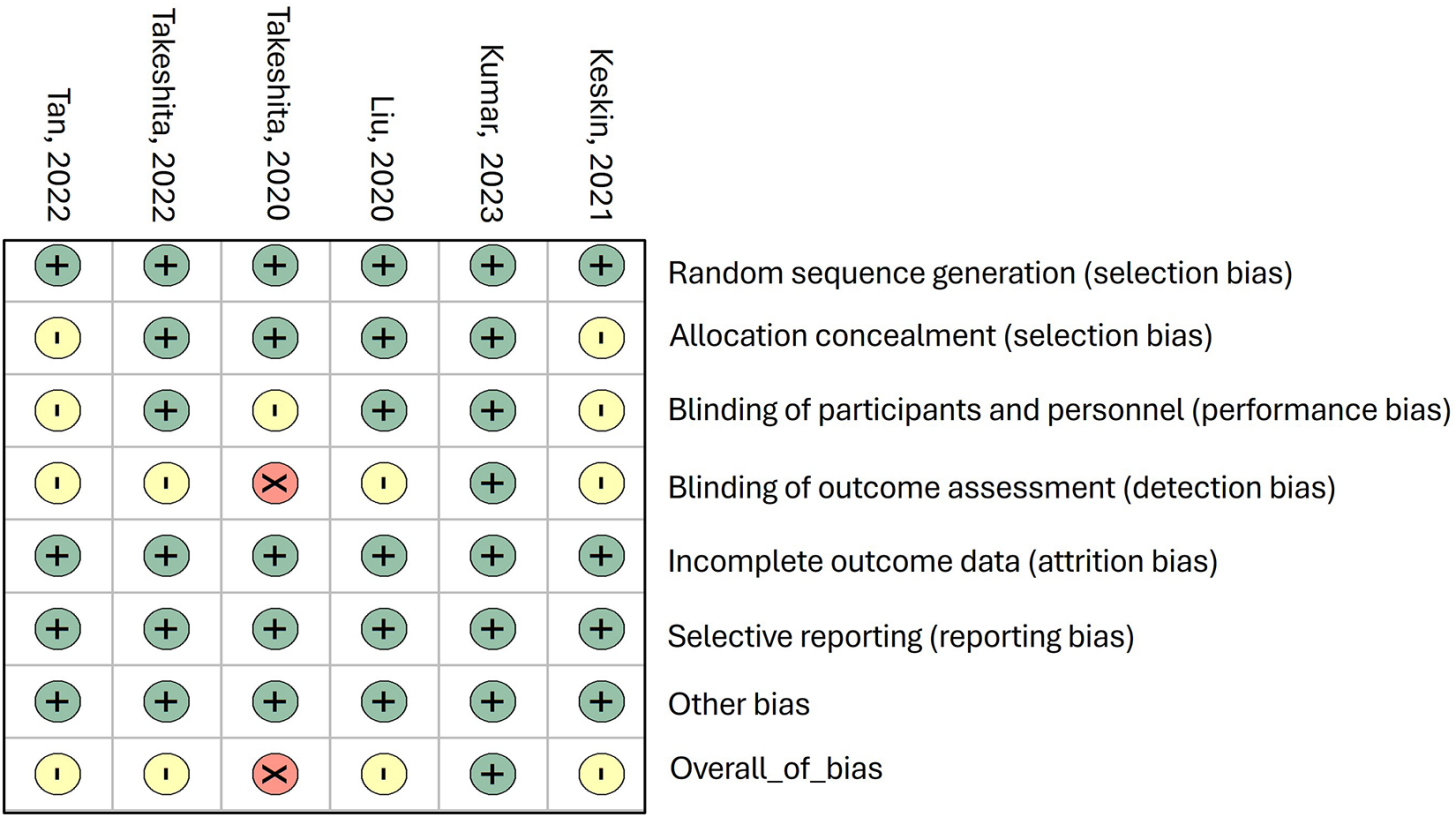
Risk of bias in the included studies.

The risk of bias within the included studies is illustrated in Figure 2.

### 3.3 Outcomes

#### 3.3.1 Catheterization success rates and cannulation time

**Figure 3:**
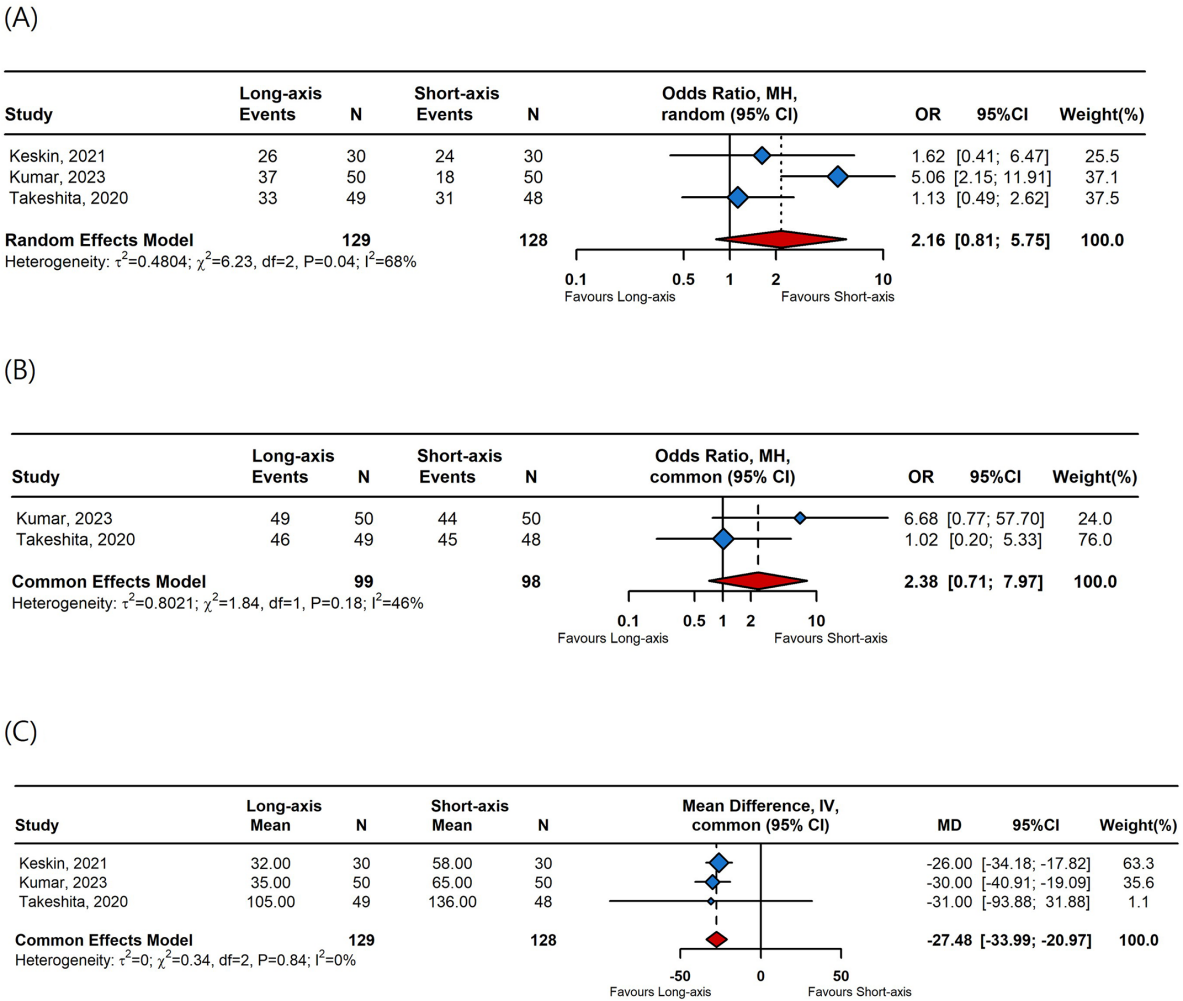
A forest plot comparing **(A)** first-attempt success rates, **(B)** overall success rates, and **(C)** cannulation time between long-axis and short-axis. OR, odds ratio; CI, confidence interval; MD, mean difference.

Data from three studies were integrated into the meta-analysis for catheterization success rates and cannulation time. No significant differences were observed in first-attempt success rates (OR 2.16, 95% CI, 0.81–5.75, Figure 3A) or overall success rates (OR 2.38, 95% CI, 0.71–7.97, Figure 3B) between the long-axis and short-axis approaches. However, the cannulation time was significantly shorter with the long-axis approach compared to the short-axis approach (MD −27.48 s, 95% CI, −33.99 to −20.97, Figure 3C).

#### 3.3.2 Catheterization complications

**Figure 4:**
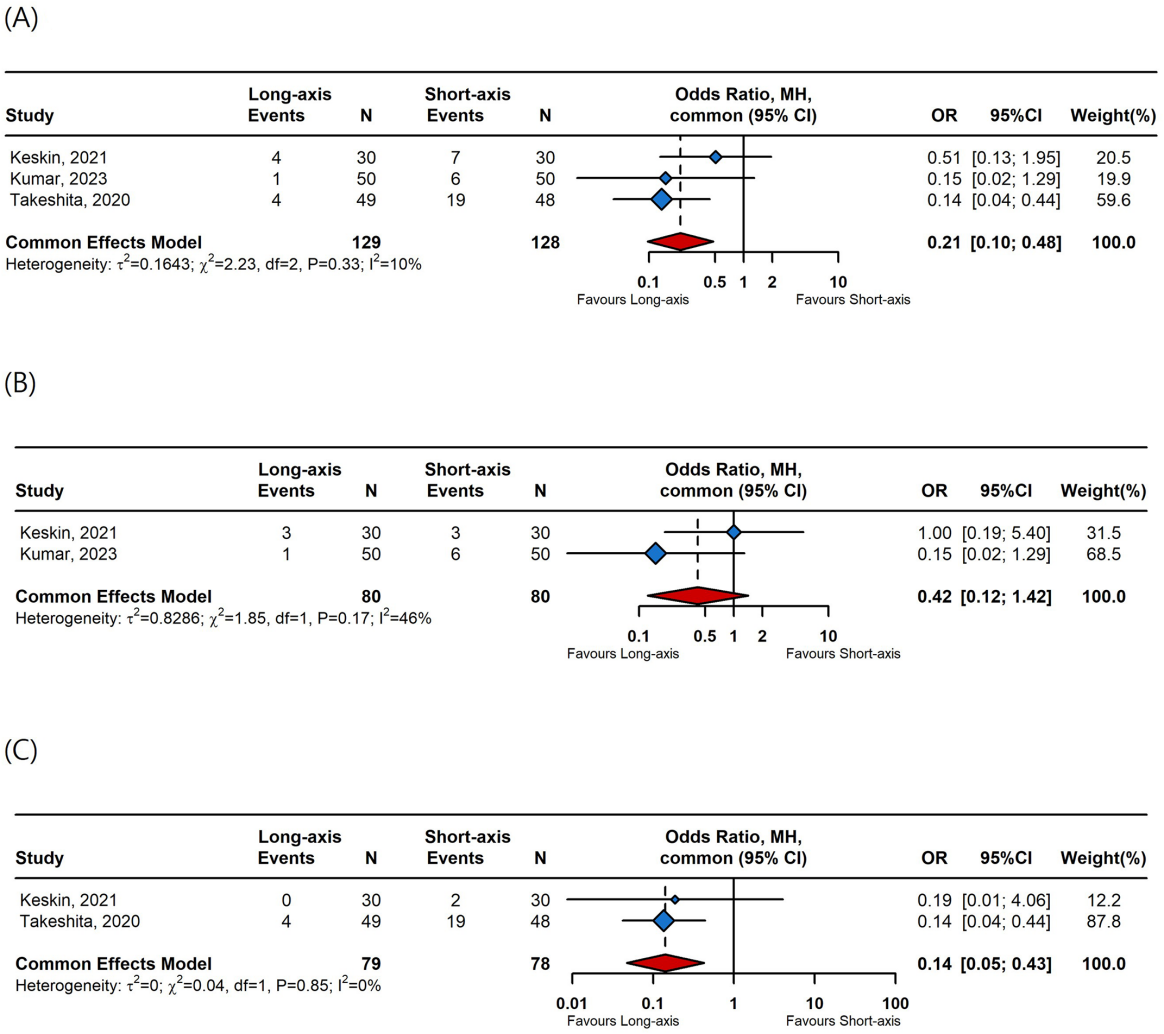
A forest plot comparing **(A)** overall complication rates, **(B)** hematoma rates, and **(C)** posterior wall puncture rates between long-axis and short-axis. OR, odds ratio; CI, confidence interval.

Data from three studies were included in the meta-analysis for complications. The long-axis approach significantly reduced overall complications (OR 0.21, 95% CI, 0.1–0.48, Figure 4A) and posterior wall punctures (OR 0.14, 95% CI, 0.05–0.43, Figure 4C) compared to the short-axis approach. However, there was no difference in hematoma incidences between the long-axis and short-axis approaches (OR 0.42, 95% CI, 0.12–1.42, Figure 4B).

## 4 Discussion

This systematic review and meta-analysis, which included 547 children undergoing CICC, demonstrates that the long-axis approach significantly reduces cannulation time and catheterization complications compared to the short-axis approach. However, it did not demonstrate significant effects on success rates.

Depending on the US probe's position relative to the vessel, US-guided CICC can be categorized into short-axis and long-axis views. Needle insertions are classified as in-plane or out-of-plane based on their visibility in the US image. The short-axis out-of-plane view provides a cross-sectional image of the vessel, enhances visualization of arterial and venous structures, reduces the risk of arterial puncture, and is simpler for less experienced physicians to learn (15, 30). However, this traditional technique lacks consistent needle tip visualization, which can lead to higher complication rates, longer cannulation times, and an increased risk of posterior wall puncture. In contrast, the long-axis in-plane view offers a longitudinal image and improves needle tip visualization (15, 16, 31). A combined technique begins with a short-axis view and rotates the probe to a long-axis (17), and has been shown to have a lower incidence of posterior wall puncture in adult patients (32).

More recently, the DNTP technique has emerged as a modification of the short-axis out-of-plane approach, aiming to improve needle tip tracking while retaining the cross-sectional vessel view. DNTP starts with a short-axis view, moves the probe away, and advances the needle until the tip is visible in the vessel lumen (18). This modification has largely replaced the traditional static out-of-plane approach in clinical practice, as it improves needle tip guidance while maintaining the benefits of short-axis imaging. As a result, the main clinical debate now lies in the choice between DNTP and in-plane approaches, as both methods provide comparable success rates while differing in operator preference and training (33, 34).

Central catheterization in children is more challenging than in adults, primarily due to distinct anatomical and physiological differences. Pediatric patients generally possess thinner, more delicate veins requiring careful needle handling and placement (35). The smaller vessel size not only complicates vein access but also increases the likelihood of complications such as puncturing the posterior wall or causing vessel trauma. Moreover, younger children with increased adiposity may experience obscured vascular lanmarks during CICC placement. Another challenge in children is their lower intravascular pressure, which can lead to vessel collapse under the weight of ultrasound transducer. Additionally, pediatric patients present unique challenges because they rarely lie still during the procedure, particularly if they are non-intubated or awake. Comfort measures, sedation, or child-friendly distraction techniques are often necessary to ensure procedural success and patient cooperation. These factors underscore the importance of selecting the most suitable and safe CICC method for pediatric patients.

Previous meta-analyses on different axis approaches of CICC primarily focused on adults (19, 20). The most recent meta-analysis in adults indicated that the short-axis approach might offer advantages such as higher first needle pass success rates, potentially reducing cannulation attempts and access time (36). However, our study demonstrated that the long-axis approach offers shorter cannulation times and fewer complications in pediatric patients. This difference could be attributed to the unique anatomical and physiological characteristics of children, which may influence the outcomes of different CICC approaches. To our knowledge, this is the first systematic review and meta-analysis in pediatrics comparing different axis approaches for US-guided CICC.

Our analysis revealed no significant difference in first-attempt or overall success rates between the long-axis and short-axis approaches, suggesting that both methods are equally effective for pediatric CICC. However, the long-axis approach significantly reduced the duration of cannulation compared to the short-axis approach. This efficiency may be attributed to the continuous visualization of the needle tip provided by the long-axis view, facilitating more accurate needle placement.

Furthermore, the long-axis approach significantly decreased overall complications and posterior wall punctures compared to the short-axis approach, making it a safer option for pediatric CICC. Continuous visualization of the needle tip during insertion likely contributes to the reduced complication rates, as it allows for more precise needle guidance and reduces the risk of accidental puncture. However, there was no significant difference in hematoma rates between the two approaches, and carotid artery puncture rates could not be analyzed due to limited results.

The DNTP approach, designed to improve needle tracking while maintaining a short-axis view, was excluded from meta-analysis due to heterogeneity of study designs but was included in the systematic review. DNTP, which is a modified short-axis approach first introduced by Clemmesen et al. for peripheral venous cannulation (37), suggests that while it may offer theoretical benefits such as improved needle tip visualization, its practical advantages in pediatric CICC over established techniques require further investigation. In adults, only a few studies have demonstrated the advantage of the DNTP method over the palpation technique in arterial cannulation (18, 38). Future studies should focus on larger sample sizes and standardized protocols to better assess the potential benefits of DNTP in pediatric CICC.

Moreover, US pre-assessment has emerged as a crucial component in central venous access procedures, offering numerous benefits for both clinicians and patients. Protocols like the Rapid Central Vein Assessment allow clinicians to evaluate vascular anatomy, vessel condition, and any anatomical variations before cannulation (39). As guidelines increasingly emphasize US guidance, integrating a standardized US pre-assessment protocol is now essential for improving clinical outcomes (40, 41).

There are also certain limitations in this study. Firstly, the number of included studies and patients was relatively small. Secondly, the heterogeneity among the included studies concerning patient populations, operator experience, and procedural protocols could affect the outcomes. Thirdly, we could not conduct meta-analysis of the combination of two approaches and the oblique approach due to limited data. Lastly, the variability in follow-up periods among studies may not adequately capture long-term complications associated with different CICC approaches. Future research should address these limitations by conducting larger, well-designed randomized controlled trials with standardized outcome measures and extended follow-up periods.

In conclusion, the long-axis approach for US-guided CICC in children significantly reduces cannulation time and catheterization complications compared to the short-axis approach. Despite the innovative nature of the DNTP technique, it did not demonstrate significant superiority in our analysis. These findings support the use of the long-axis approach in pediatric CICC procedures, although there were no differences in success rates. Further research is needed to refine these techniques and explore their application in diverse clinical settings.

## Data Availability

The data analyzed in this study is subject to the following licenses/restrictions: the dataset used in this study is derived from previously published randomized controlled trials (RCTs) and is publicly available through the original publications. As such, the dataset is subject to the restrictions and limitations imposed by those original studies, including access to full datasets, specific study methodologies, and any proprietary or ethical restrictions placed by the original authors. The data extracted and analyzed in this meta-analysis are limited to what was reported in these published studies, and no additional or raw data were generated or accessed beyond the scope of these publications. Therefore, the dataset is not directly available through this article but can be accessed through the referenced studies. Requests to access these datasets should be directed to inkyung9233@hanmail.net.
