# Supplementary material for: Different axis approaches for ultrasound-guided centrally inserted central catheterization in children: a systematic review and meta-analysis of randomized controlled trials
Source: Front Surg. 2025 Feb 24;12:1481975. doi: 10.3389/fsurg.2025.1481975 (PMC11891167; doi:10.3389/fsurg.2025.1481975)
Supplement: Supplementary file 1 [file Datasheet1.docx]

Supplement 1. Details of search strategy

| **DB** | **Search** | **Search terms** | **Results** |
| --- | --- | --- | --- |
| **PubMed** | #1 | "Ultrasonography, Interventional"[Mesh] | 32,785 |
|  | #2 | "Ultrasonography, Interventional"[TW] OR "Interventional Ultrasonography"[TW] OR "Ultrasound, Interventional"[TW] OR "Interventional Ultrasound"[TW] OR "Ultrasonography, Intravascular"[TW] OR "Intravascular Ultrasonography"[TW] OR "Ultrasound-Guided"[TW] OR "Ultrasound guidance"[TW] | 57,923 |
|  | #3 | "Ultrasonography"[Mesh] | 496,774 |
|  | #4 | "Ultrasonography"[TW] OR "Echotomography"[TW] OR "Ultrasonic Imaging"[TW] OR "Imaging, Ultrasonic"[TW] OR "Sonography, Medical"[TW] OR "Medical Sonography"[TW] OR "Ultrasound Imaging"[TW] OR "Imagings, Ultrasound"[TW] OR "Imaging, Ultrasound"[TW] OR "Ultrasonographic Imaging"[TW] OR "Imagings, Ultrasonographic"[TW] OR "Imaging, Ultrasonographic"[TW] OR "Ultrasonographic Imagings"[TW] OR "Echography"[TW] OR "Diagnostic Ultrasound"[TW] OR "Diagnostic Ultrasounds"[TW] OR "Ultrasound, Diagnostic"[TW] OR "Ultrasounds, Diagnostic"[TW] OR "Echotomography, Computer"[TW] OR "Computer Echotomography"[TW] OR "Tomography, Ultrasonic"[TW] OR "Ultrasonic Tomography"[TW] OR "Diagnosis, Ultrasonic"[TW] OR "Diagnoses, Ultrasonic"[TW] OR "Ultrasonic Diagnoses"[TW] OR "Ultrasonic Diagnosis"[TW] | 384,267 |
|  | **#5 Combine** | **#1 OR #2 OR #3 OR #4** | **574,514** |
|  | #6 | "Catheterization, Central Venous"[Mesh] | 17,294 |
|  | #7 | "Catheterization, Central Venous"[TW] OR "Central Venous Catheterization"[TW] OR "Catheterizations, Central Venous"[TW] OR "Central Venous Catheterizations"[TW] OR "Venous Catheterizations, Central"[TW] OR "Venous Catheterization, Central"[TW] OR "Catheterization, Central"[TW] OR "Catheterizations, Central"[TW] OR "Central Catheterizations"[TW] OR "Central Catheterization"[TW] | 18,157 |
|  | **#8 Combine** | **#6 OR #7** | **18,157** |
|  | **#9 Combine** | **#5 AND #8** | **2,646** |
|  | **#10 Limit** | **#9 AND (allchild[Filter])** | **696** |
|  | **#11 Limit** | **#10 AND (randomizedcontrolledtrial[Filter])** | **60** |
|  | **#12 Limit** | **#11 NOT ("animals"[MeSH] NOT "Humans"[MeSH])** | **60** |
|  |  |  |  |
|  |  |  |  |
| **DB** | **Search** | **Search terms** | **Results** |
| **EMBASE** | #1 | "interventional ultrasonography"/exp | 6,557 |
|  | #2 | "Ultrasonography, Interventional":ti,ab,kw,de OR "Interventional Ultrasonography":ti,ab,kw,de OR "Ultrasound, Interventional":ti,ab,kw,de OR "Interventional Ultrasound":ti,ab,kw,de OR "Ultrasonography, Intravascular":ti,ab,kw,de OR "Intravascular Ultrasonography":ti,ab,kw,de OR "Ultrasound-Guided":ti,ab,kw,de OR "Ultrasound guidance":ti,ab,kw,de | 73,441 |
|  | #3 | "echography"/exp | 1,098,792 |
|  | #4 | "Ultrasonography":ti,ab,kw,de OR "Echotomography":ti,ab,kw,de OR "Ultrasonic Imaging":ti,ab,kw,de OR "Imaging, Ultrasonic":ti,ab,kw,de OR "Sonography, Medical":ti,ab,kw,de OR "Medical Sonography":ti,ab,kw,de OR "Ultrasound Imaging":ti,ab,kw,de OR "Imagings, Ultrasound":ti,ab,kw,de OR "Imaging, Ultrasound":ti,ab,kw,de OR "Ultrasonographic Imaging":ti,ab,kw,de OR "Imagings, Ultrasonographic":ti,ab,kw,de OR "Imaging, Ultrasonographic":ti,ab,kw,de OR "Ultrasonographic Imagings":ti,ab,kw,de OR "Echography":ti,ab,kw,de OR "Diagnostic Ultrasound":ti,ab,kw,de OR "Diagnostic Ultrasounds":ti,ab,kw,de OR "Ultrasound, Diagnostic":ti,ab,kw,de OR "Ultrasounds, Diagnostic":ti,ab,kw,de OR "Echotomography, Computer":ti,ab,kw,de OR "Computer Echotomography":ti,ab,kw,de OR "Tomography, Ultrasonic":ti,ab,kw,de OR "Ultrasonic Tomography":ti,ab,kw,de OR "Diagnosis, Ultrasonic":ti,ab,kw,de OR "Diagnoses, Ultrasonic":ti,ab,kw,de OR "Ultrasonic Diagnoses":ti,ab,kw,de OR "Ultrasonic Diagnosis":ti,ab,kw,de | 637,450 |
|  | **#5 Combine** | **#1 OR #2 OR #3 OR #4** | **1,179,330** |
|  | #6 | "central venous catheterization"/exp | 10,600 |
|  | #7 | "Catheterization, Central Venous":ti,ab,kw,de OR "Central Venous Catheterization":ti,ab,kw,de OR "Catheterizations, Central Venous":ti,ab,kw,de OR "Central Venous Catheterizations":ti,ab,kw,de OR "Venous Catheterizations, Central":ti,ab,kw,de OR "Venous Catheterization, Central":ti,ab,kw,de OR "Catheterization, Central":ti,ab,kw,de OR "Catheterizations, Central":ti,ab,kw,de OR "Central Catheterizations":ti,ab,kw,de OR "Central Catheterization":ti,ab,kw,de | 11,361 |
|  | **#8 Combine** | **#6 OR #7** | **11,519** |
|  | **#9 Combine** | **#5 AND #8** | **2,426** |
|  | **#10 Limit** | **#9 AND ([newborn]/lim OR [infant]/lim OR [child]/lim OR [adolescent]/lim)** | **434** |
|  | **#11 Limit** | **#10 AND [randomized controlled trial]/lim** | **36** |
|  | **#12 Limit** | **#11 NOT ('animal'/exp NOT 'human'/exp)** | **36** |
|  |  |  |  |
|  |  |  |  |
| **DB** | **Search** | **Search terms** | **Results** |
| **Cochrane Library** | #1 | [mh "Ultrasonography, Interventional"] | 3,554 |
|  | #2 | "Ultrasonography, Interventional":ti,ab,kw OR "Interventional Ultrasonography":ti,ab,kw OR "Ultrasound, Interventional":ti,ab,kw OR "Interventional Ultrasound":ti,ab,kw OR "Ultrasonography, Intravascular":ti,ab,kw OR "Intravascular Ultrasonography":ti,ab,kw OR "Ultrasound-Guided":ti,ab,kw OR "Ultrasound guidance":ti,ab,kw | 15,020 |
|  | #3 | [mh "Ultrasonography"] | 19,360 |
|  | #4 | "Ultrasonography":ti,ab,kw OR "Echotomography":ti,ab,kw OR "Ultrasonic Imaging":ti,ab,kw OR "Imaging, Ultrasonic":ti,ab,kw OR "Sonography, Medical":ti,ab,kw OR "Medical Sonography":ti,ab,kw OR "Ultrasound Imaging":ti,ab,kw OR "Imagings, Ultrasound":ti,ab,kw OR "Imaging, Ultrasound":ti,ab,kw OR "Ultrasonographic Imaging":ti,ab,kw OR "Imagings, Ultrasonographic":ti,ab,kw OR "Imaging, Ultrasonographic":ti,ab,kw OR "Ultrasonographic Imagings":ti,ab,kw OR "Echography":ti,ab,kw OR "Diagnostic Ultrasound":ti,ab,kw OR "Diagnostic Ultrasounds":ti,ab,kw OR "Ultrasound, Diagnostic":ti,ab,kw OR "Ultrasounds, Diagnostic":ti,ab,kw OR "Echotomography, Computer":ti,ab,kw OR "Computer Echotomography":ti,ab,kw OR "Tomography, Ultrasonic":ti,ab,kw OR "Ultrasonic Tomography":ti,ab,kw OR "Diagnosis, Ultrasonic":ti,ab,kw OR "Diagnoses, Ultrasonic":ti,ab,kw OR "Ultrasonic Diagnoses":ti,ab,kw OR "Ultrasonic Diagnosis":ti,ab,kw | 26,175 |
|  | **#5 Combine** | **#1 OR #2 OR #3 OR #4** | **41,576** |
|  | #6 | [mh "Catheterization, Central Venous"] | 1,153 |
|  | #7 | "Catheterization, Central Venous":ti,ab,kw OR "Central Venous Catheterization":ti,ab,kw OR "Catheterizations, Central Venous":ti,ab,kw OR "Central Venous Catheterizations":ti,ab,kw OR "Venous Catheterizations, Central":ti,ab,kw OR "Venous Catheterization, Central":ti,ab,kw OR "Catheterization, Central":ti,ab,kw OR "Catheterizations, Central":ti,ab,kw OR "Central Catheterizations":ti,ab,kw OR "Central Catheterization":ti,ab,kw | 1,494 |
|  | **#8 Combine** | **#6 OR #7** | **1,494** |
|  | **#9 Combine** | **#5 AND #8** | **388** |
|  | **#10 Limit** | **#9 AND ([mh "Child"] OR [mh "Child, Preschool"] OR [mh "Infant"] OR [mh "Infant, Newborn"])** | **71** |
|  | **#11 Limit** | **#10 NOT ([mh "animals"] NOT [mh "Humans"])** | **71** |
